# Dynamics of the Salivary Microbiome During Different Phases of Crohn's Disease

**DOI:** 10.3389/fcimb.2020.544704

**Published:** 2020-10-06

**Authors:** Tianyu Zhang, Masood ur Rehman Kayani, Liwen Hong, Chen Zhang, Jie Zhong, Zhengting Wang, Lei Chen

**Affiliations:** ^1^Department of Gastroenterology, Ruijin Hospital Affiliated to Shanghai Jiao Tong University School of Medicine, Shanghai, China; ^2^Center for Microbiota and Immunological Diseases, Shanghai General Hospital, Shanghai Institute of Immunology, School of Medicine, Shanghai Jiao Tong University, Shanghai, China

**Keywords:** Crohn's disease, salivary micro biome, active stage, differential abundance, functional annotation, metabolic pathways

## Abstract

Crohn's disease is a chronic disorder that typically affects the gastrointestinal tract. The increased incidence in the recent years, especially in Asian countries, prompts for performing studies and gain newer insights into the etiology and pathogenesis of the disease. Among other causative factors, gut microbiome and its cross-talk with the salivary microbiome is a known factor that has a plausible role in the pathogenesis of Crohn's disease. The gut microbiome has been extensively studied, however, the salivary microbiome and its dynamics during different phases of this disease remain understudied. In this study, we obtained saliva samples from the patients during active and remission phases of the disease and compared them with control samples and highlighted the differences in taxonomic as well as predicted functional pathways among them. Our results indicated that the α and β diversities were significantly lower during the active phase in contrast with remission phase and healthy samples. In general, *Firmicutes* were most abundant among the three sample groups, followed by *Bacteroidetes* and *Proteobacteria*. Genus level distribution highlighted *Streptococcus, Neisseria, Prevotella, Haemophilus*, and *Veillonella* as the five most abundant taxa. Differential abundance analysis of the three sample groups identified significant enrichment of 30 bacterial taxa in the active phase that included g_*Prevotella*, f_*Prevotellaceae*, and p_*Bacteroidetes*. Furthermore, remission phase and control also exhibited significant enrichment of 24 and 22 bacterial taxa, respectively. Eleven differentially abundant pathways were also identified, four were significantly enriched in healthy controls whereas other seven were significantly enriched in active phase of the disease. Several important pathways, such as ribosome biogenesis and Energy metabolism were depleted in the active phase. Our study has highlighted several taxa and functional categories that could be implicated with the onset of Crohn's disease and thus have the potential to serve as biomarkers of the active disease. However, these findings require further validation through functional studies in the future.

## Introduction

Crohn's disease (CD) is a chronic gastrointestinal (GI) inflammatory disorder that can affect any segment of the GI tract (Kaser et al., 2010). The incidence of CD has increased rapidly during the recent years in Asia (Prideaux et al., 2012; Kaplan and Ng, 2017). The clinical activity of CD is typically judged using Crohn's Disease Activity Index (CDAI), a scoring system that is widely adapted based on patients' clinical symptoms. Alternatively, Montreal classification is also used to subset CD patients into different subgroups according to age of onset, disease location and disease behavior. Montreal classification is often very helpful for clinical treatment and follow-up. The precise etiology and pathogenesis of CD remain unclear (Khor et al., 2011). The most accepted hypothesis is that complex interactions between genetics, environmental factors, and the host immune system lead to aberrant immune responses and chronic intestinal inflammation. The gut microbiome has physiological functions associated with nutrition, the immune system, and defense of the host which indicated its possible role in the pathogenesis of CD (Honda and Littman, 2012; Goldsmith and Sartor, 2014; Sheehan et al., 2015).

The fecal microbiota of CD patients had been extensively studied. Many studies had showed that the fecal microbiota in CD had altered composition and function compared with that in healthy people, which is known as dysbiosis (Andoh et al., 2007; Fujimoto et al., 2013; Takahashi et al., 2016; Sartor and Wu, 2017; Nishino et al., 2018). These changes could be summarized by decrease with anti-infammatory capacities and the increase with inflammatory capacities [13-14]. The most consistent changes were a reduction in the diversity of gut microbiota and the lower abundance of *Firmicutes* (Manichanh et al., 2006; Frank et al., 2007; Peterson et al., 2008). It had been showed that *F. prausnitzii, Blautia faecis, Roseburia inulinivorans, Ruminococcus torques*, and *Clostridium lavalense* were decreased in CD patients when compared to healthy subjects and that the number of *F. prausnitzii* was correlated with the relapse risk of ileal CD after surgery (Fujimoto et al., 2013; Takahashi et al., 2016).

Oral mucosal inflammation is well-studied in patients with IBD with a reported prevalence of 0.5–80% in CD patients (Rowland et al., 2010). Symptoms could vary from mild and non-specific inflammation such as minor aphthous lesions, mucogingivitis, and angular cheilitis to more specific findings, such as cobblestoning, deep linear ulcerations, and more severe orofacial granulomatosis (OFG) (Katz et al., 2003; Ojha et al., 2007). Moreover, oral mucosal lesions may occur much earlier before the onset of intestinal symptoms (Galbraith et al., 2005). However, we have limited knowledge of characteristics of saliva microbiota in CD patients, and the correlation of oral inflammation and the pathogenesis of CD. Docktor et al. had found that a significant decrease in overall diversity in the oral microbiome of pediatric CD compared with the healthy (Docktor et al., 2012). Said et al. showed that some dominant genera, *Streptococcus, Prevotella, Neisseria, Haemophilus, Veillonella, and Gemella*, were found to largely contribute to dysbiosis observed in the salivary microbiota of IBD patients (Said et al., 2014). But as far as we know, the differences in saliva microbiota during active and remission as well as in patients with different disease location and behavior phases remain understudied.

In this study, we explored the characteristics of saliva microbiota of CD patients using samples from active and remission phases, and healthy controls. We also identified significantly differentially abundant taxa and metabolic pathways in CD patients that have the potential to serve as biomarkers of the disease onset.

## Materials and Methods

### Patients and Control Recruitment and Sample Collection

From September 2019 to December 2019, patients diagnosed with CD in Ruijin Hospital, Shanghai, China were recruited in our study. Control subjects were enrolled from healthy volunteers in our hospital, people with a history of chronic inflammatory disorder including IBD and current gastrointestinal symptoms were excluded. The diagnosis of CD was based on an integrated judgement of clinical symptoms, endoscopic characteristics, radiological findings, and histological features. Subjects were excluded from this study if they had antibiotics exposure in 1 month before sampling. The study was approved by the institutional ethics board of our hospital. Informed consent was acquired from all the enrolled subjects. 2 mL saliva was collected from patients or volunteers in a sterile container and immediately placed on ice, and transferred within 3 h to a storage freezer at −70°C.

### Clinical Data Collection

Clinical data included the patient's age, gender, duration of symptom, smoking history, and bowel surgery. Samples from patients with severe oral problems, such as dental caries and periodontal disease, were excluded from the study. Montreal classification of each patient including age at diagnosis, location, disease behavior, and perianal disease were recorded. Crohn's Disease Activity Index (CDAI) > 150 was regarded as active phase while CDAI < 150 or CDAI = 150 as remission phase.

### DNA Extraction and 16S rRNA Sequencing

Microbial DNA was extracted from saliva samples using the DNA extraction Kit (QIAamp DNA Mini Kit, QIAGEN, Germany) according to manufacturer's protocols. The V3–V4 region of the bacteria 16S ribosomal RNA genes were amplified by PCR (95°C for 3 min, followed by 30 cycles at 98°C for 20 s, 58°C for 15 s, and 72°C for 20 s and a final extension at 72°C for 5 min) using primers 341F 5′-CCTACGGGRSGCAGCAG)-3′ and 806R 5′-GGACTACVVGGGTATCTAATC-3′. PCR reactions were performed in 30 μL mixture containing 15 μL of 2 × KAPA Library Amplification ReadyMix, 1 μL of each primer (10 μM), 50 ng of template DNA and ddH_2_O.

Amplicons were extracted from 2% agarose gels and purified using the AxyPrep DNA Gel Extraction Kit (Axygen Biosciences, Union City, CA, U.S.) according to the manufacturer's instructions and quantified using Qubit^®^ 2.0 (Invitrogen, U.S.). After preparation of library, these tags were sequenced on MiSeq platform (Illumina, Inc., CA, USA) for paired end reads of 250 bp, which were overlapped on their three ends for concatenation into original longer tags. DNA extraction, Library construction and sequencing were conducted at Realbio Genomics Institute (Shanghai, China).

### Taxonomic and Functional Analysis

Tags, trimmed of barcodes and primers, were further checked on their rest lengths and average base quality. 16S tags were restricted between 220 bp and 500 bp such that the average Phred score of bases was no worse than 20 (Q20) and no more than three ambiguous N. The copy number of tags was enumerated and redundancy of repeated tags was removed. Only the tags with frequency more than 1, which tend to be more reliable, were clustered into OTUs, each of which had a representative tag. Operational Taxonomic Units (OTUs) were clustered with 97% similarity using UPARSE (Edgar, 2013) and chimeric sequences were identified and removed using Userach v7.0 (Edgar, 2010). Each representative tags were assigned to a taxon by RDP Classifier against the RDP database (Wang et al., 2007) using confidence threshold of 0.8. OTU profiling and alpha/beta diversity analyses were performed using QIIME (Caporaso et al., 2010). The functional analysis, for predicting the pathways, was performed using PICRUSt (Langille et al., 2013). Briefly, the OTU table was normalized using the *normalize_by_copy_number.py* script, followed by functional predictions of KEGG Ortholog (KOs) using the *predict_metagenomes.py* script. The predicted KOs were collapsed to KEGG pathways using the *categorize_by_function.py* script. All of the three mentioned scripts are included in the standard PICRUSt package. The identification of differentially abundant Taxa and metabolic pathways was performed using the Linear discriminant analysis Effect Size (LEfSe) (Segata et al., 2011) using the logarithmic LDA score for discriminative features was set to >2.0 as threshold for differentially taxa and LDA score of >2.5 for metabolic pathways.

## Results

### Sample Characteristics

In total, we collected and sequenced 91 samples from different patients which included 29 patients from active phase of CD, and 31 patients from remission phase of CD and 31 healthy controls. The active phase samples included 3 L1, 4 L2, 21 L3, and 1 L4 samples, as categorized on the basis of Montreal classification. In contrast, Remission phase included 8 L1, 6 L2, 14 L3, and 4 L4 samples. The metadata of these samples is provided in Supplementary Table 1.

After preprocessing the raw reads, minimum and maximum number of high quality (≥Q20) was 38,944 and 29,240, respectively. Furthermore, the minimum proportion of reads passing the Q20 and Q30 quality thresholds was 97.12 and 91.58%, respectively. Read length distribution indicated that the average read length was ≥ 400 bp, whereas the maximum number of reads (> 2.5M reads) was in the range of 420–440 bp. Only few reads were either longer than 440 bp or shorter than 400 bp. The read length distributions are indicated in Supplementary Figure 1A.

### Alpha and Beta Diversity

**Figure 1:**
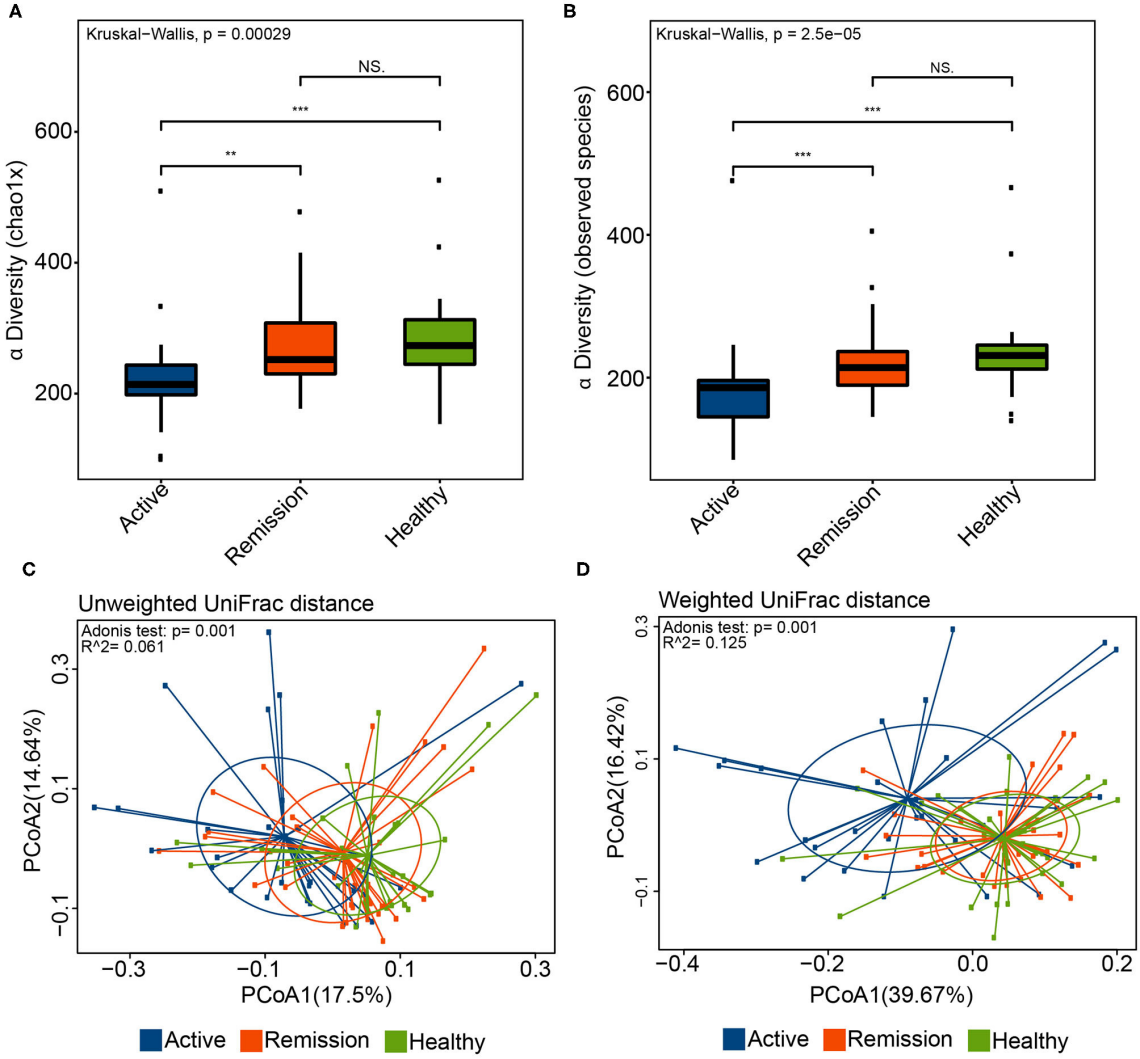
Alpha and Beta diversity in the oral microbiome of IBD patients and healthy controls. **(A)** Chao1 diversity index in the three sample groups. **(B)** Boxplot indicating the number of observed species in the three sample categories. **(C)** Principle coordinate analysis (PCoA) using the unweighted UniFrac distance and Adonis test. Blue color represents samples from active phase, orange indicates remission phase while green color indicates healthy samples. **(D)** PCoA using the weighted UniFrac distance and Adonis test. ^**^ and ^***^ correspond to *P* values < 0.01 and 0.001, respectively.

The comparative analysis of alpha diversity highlighted significant differences among the three sample categories. The chao1 index, reflective of the estimated number of species, indicated that the alpha diversity was significantly lower in the active phase in contrast with the remission phase (*P* = 0.0015) as well as the control group (*P* = 0.00011) (Figure 1A). Good's coverage was also significantly different between active/remission phases (*P* = 0.016) as well as between active phase of disease and control (*P* = 0.00039) (Supplementary Figure 1B). Furthermore, there was no significant difference between the remission phase and control in terms of chao1 and Good's coverage (*P* = 0.39 and 0.2, respectively). The number of observed species was significantly lower in the active disease phase in contrast with remission phase (*P* = 0.00058) and control (*P* = 2.5e-05) (Figure 1B). Similarly, the phylogenetic diversity (PD) also showed significant decrease in the active phases in contrast with the control group (*P* = 0.0027) and remission phase (*P* = 2e-04) of disease. No significant differences were observed among the three groups using Shannon and Simpson indices (Supplementary Figures 1C–E). The alpha diversity (Chao1, Goods coverage, PD) did not significantly differ among different Montreal levels of disease (i.e., L1–L4). The only significant difference was observed between the control and L3 category of samples using chao1 index (*P* < 0.0026), Good's coverage (*P* < 0.0018), observed species (*P* < 0.00048), and PD (*P* < 0.0036). These results are shown in Supplementary Figures 2A–C.

Principle coordinate analysis (PCoA) using the unweighted and weighted UniFrac distances also indicated that the overall oral microbiomes differed significantly among the three groups (Figures 1C,D). Although, the *p*-values obtained using Adonis test for both distances (*P* = 0.001) were significant, the *R*^2^ values were indicative of small variance among the groups (i.e., *R*^2^ 0.061 and 0.125 for unweighted and weighted UniFrac distances, respectively). Based on these β-diversities, cluster analysis revealed that majority of control samples clustered with samples from remission phase whereas most of the oral microbiome samples from the active disease patients clustered separately. Adonis test for the four different levels (L1–L4) and control, using the weighted and unweighted UniFrac distances, did not indicate presence of any significant differences in the samples (*P* > 0.05, Supplementary Figures 2D,E).

### OTU and Distribution of Common Taxa in the Three Sample Groups

**Figure 2:**
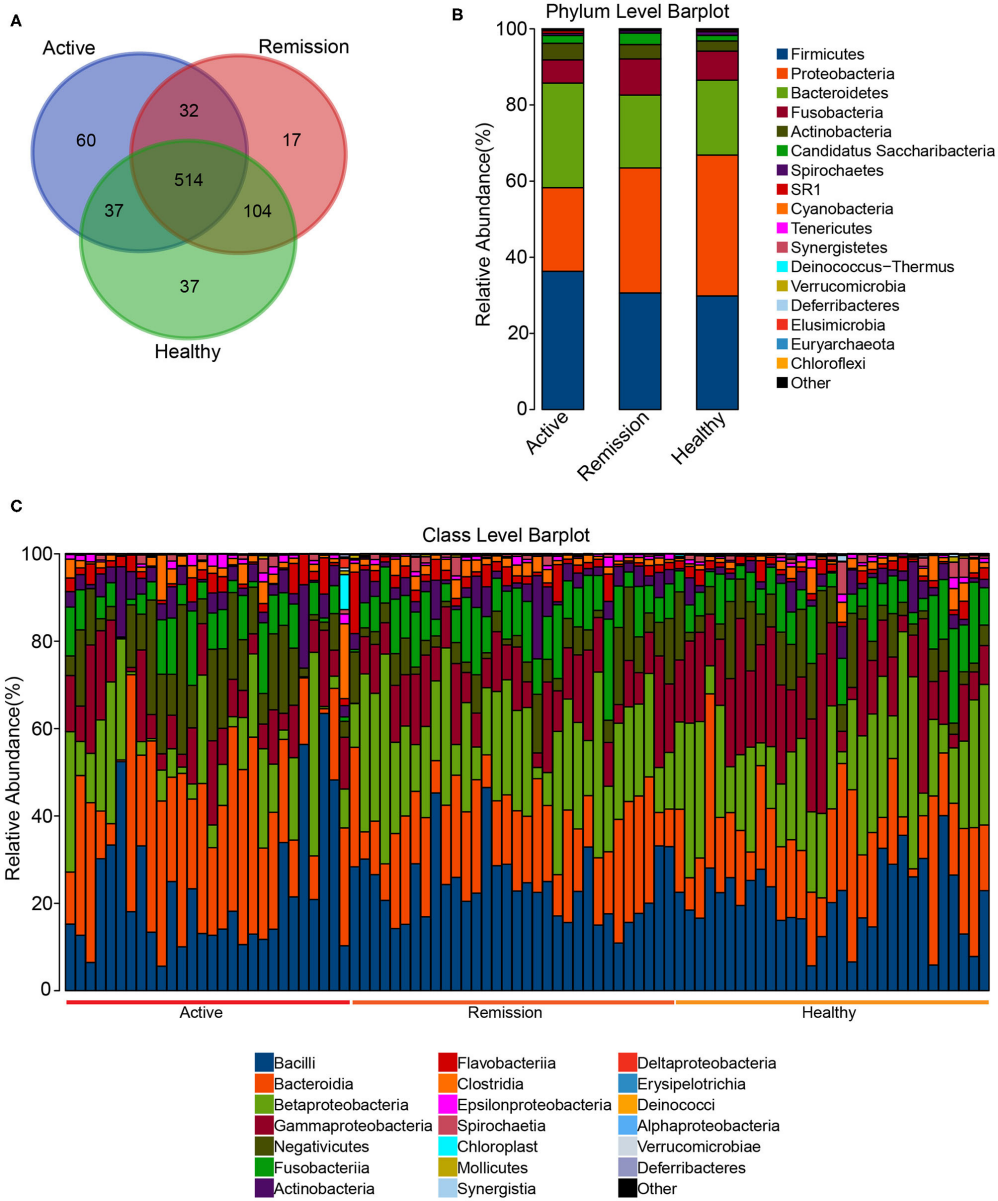
OTU count and Taxonomic distribution in the oral microbiome of CD patients and healthy controls. **(A)** Venn diagram indicating the overlap of OTUs in the three categories. **(B)** Barplot of the relative abundances of different taxa at phylum level. **(C)** Barplot of the relative abundances of different taxa at Class level among all the samples.

The total of number of predicted OTUs across all samples was 794 with a minimum and maximum of 85 and 476, respectively. Among these 794 OTUs, the three categories of samples shared 514 among them, 60 were exclusively identified in the active phase, 17 in the remission phase and 37 in the control samples. Furthermore, active phase and control shared 37 common OTUs whereas control and remission phases showed a much higher number of common OTUs i.e., 104 (Figure 2A). The taxonomic classification of OTUs indicated that all 794 OTUs were classifiable at Kingdom level, 759 at Phylum level, 721 at Class, 710 at Order, 662 at Family, and 468 at Genus level (Table 1). The Phylum level distribution indicated that *Firmicutes* were highly abundant in all three types of samples. Their abundance was slightly lower in control (29.8%) and remission phase (30.6%) in comparison with active phase of disease (~36.3%). Similarly, *Bacteroidetes* were also highly abundant with relative abundances of 19.6, 19.1, and 27.4% in control, remission phase and active phase, respectively. In contrast, *Proteobacteria* were highly abundant in control and remission phase (37 and 32.8%, respectively) while showed lower abundance in active phase (~22%). Other noticeable taxa included members of *Fusobacteria* and *Actinobacteria*. Archaea were also identified but in very low relative abundance (0.0004–0.008%) and mainly represented by *Euryarchaeota* (Figure 2B). On class level, the most abundant taxa included *Bacilli, Bacteroidia, Betaproteobacteria, Gammaproteobacteria*, and *Negativicutes*. The relative abundance of *Bacteroidia* (25.44%) was higher in active phase of disease in contrast with remission phase (17.5%) and control (18.2%). *Bacilli* showed similar relative abundances in the three sample categories i.e., 20.9–23%. Furthermore, *Betaproteobacteria* were more abundant in control and samples from remission phase (20.6 and 21.6%, respectively) in contrast with active phase (12.1%). *Gammaproteobacteria* were most abundant in control (16.3%), followed by remission (11.6%) and active phases (9.3%). Similarly, *Negativicutes* were identified in higher proportion in active phase of disease (11.5%), followed by control (7.72%) and remission phase (5.8%). Additionally, *Fusobacteria, Actinobacteria, Flavobacteria*, and *Clostridia* were also among the identified taxa at class level (Figure 2C). Order and Family level distributions are shown in Supplementary Figures 3A,B.

**Table 1:** General statistics of the predicted OTUs in the enrolled samples.

**Statistics**	**Count**
No. of OTUs	794
Assigned to Kingdom	794
Assigned to Phylum	759
Assigned to Class	721
Assigned to Order	710
Assigned to Family	662
Assigned to Genus	468
Assigned to Species	0
Min no. of OTUs per sample	85
Max no. of OTUs per sample	476
Mean no. of OTUs per sample	214.802
SD of the OTUs per sample	63.5

**Figure 3:**
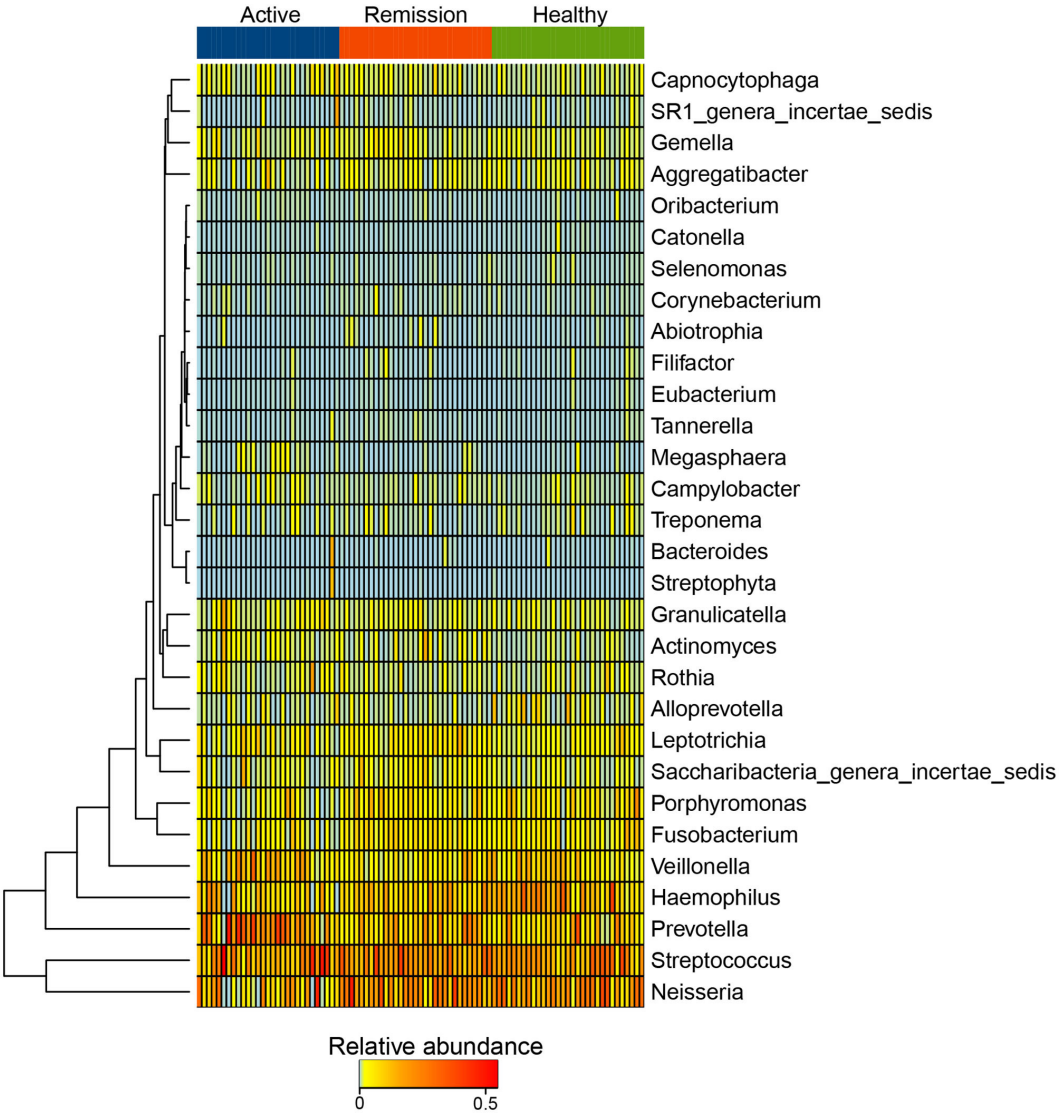
Heatmap of the distribution of 25 most abundant taxa at Genus level in active phase, remission phase, and healthy controls.

*Streptococcus, Neisseria, Prevotella, Haemophilus*, and *Veillonella* were the five most abundant genus-level taxa identified among the three categories of samples. *Streptococcus* showed similar abundance (18.2–20.1%) in the three groups, *Neisseria* was decreased in active phase (10%) in contrast with control (18.7%) and remission phase (19.8%). *Prevotella* were more abundant in the active phase (19.9%) while showed lower proportions in both the remission phase (10.4%) and controls (8.82%) (Supplementary Figure 3C, Supplementary Table 2). Based on the relative abundances of genera, cluster analysis indicated that majority of the samples from control and remission phase were clustered together. However, certain active phase samples were clustered with the remission phase and C samples as indicated in Supplementary Figure 4. The abundance profile of *Streptococcus, Neisseria, Prevotella, Haemophilus*, and *Veillonella* among all samples was quite different from the rest of the taxa, which is reflected by their distant clustering from the other genera (Figure 3). Other taxa formed sub-clusters which included genera with mixed relative abundances (i.e., low to intermediate). For example, *Capnocytophaga, SR1_genera_incertae_sedis, Gemella* and *Aggregatibacter* demonstrated mixed relative abundance and were sub-clustered. Another sub-cluster included mostly the less abundant taxa such as *Oribacterium, Selenomonas, Corynebacterium, Abiotrophia*, and *Eubacterium* (Figure 3).

### Identification of Differentially Abundant Taxa

**Figure 4:**
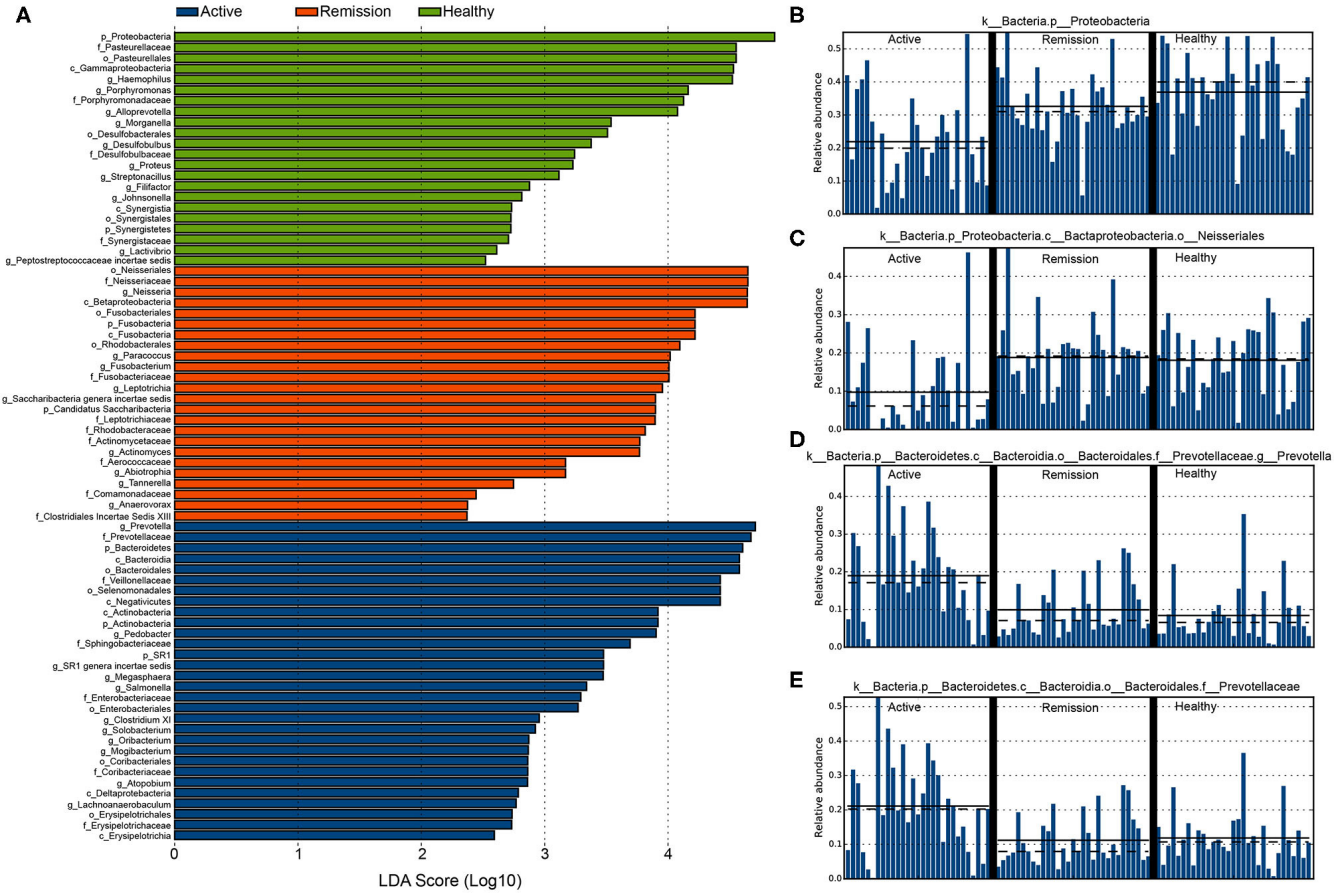
Differentially abundant taxa identified among the three categories of samples using LEfSe. **(A)** The highly enriched and significantly differentially abundant taxa in the three categories of samples (LDA score > 2 and α < 0.05). **(B)** Distribution of p__*Proteobacteria* in the three categories of samples. **(C)** o__*Neisseriales*. **(D)** g__*Prevotella*. **(E)** f__*Prevotellaceae*.

We further performed differential abundance analysis using linear discriminant analysis (LDA) with effect size measurements (LEfSe) to identify taxa that were significantly associated with active/remission phase and health. In control samples, 22 taxa were significantly (α <0.05) more abundant with an LDA score > 2. These included an unclassified member of the phylum Proteobacteria (p_*Proteobacteria*), f_*Pasterurellaceae*, an unclassified *Gammaproteobacteria* (c_*Gammaproteobacteria*), g_*Haemophilus*, and f_*Porphyromonadaceae*. Furthermore, several bacterial taxa that could be classified to genus level, including g_*Alloprevotella*, g_*Morganella*, g_*Desulfobulbus*, g_*Proteus*, g_*Filifactor*, g_*Johnsonella*, and g_*Lactivibrio* were also significantly more enriched in control samples (Figure 4A and Supplementary Figure 5A). The histogram of distribution of p_*Proteobacteria* in all three categories of samples is shown in Figure 4B.

Active phase of the disease was represented by 30 significantly enriched bacterial taxa, eight of which showed an LDA score > 4. Among these, g_*Prevotella* showed highest differential abundance, their average relative abundance among all samples in the three categories is shown in Figure 4D. Other bacterial taxa (LDA > 4) included f_*Prevotellaceae* (Figure 4E), p_*Bacteroidetes, Bacteroidia*, f_*Veillonellaceae*, o_*Selenomonadales*, and c_*Negativitcutes*. active phase samples were also enriched with g_*Pedobacter*, g_*Megasphaera*, g_*Salmonella*, g_*Clostridium XI*, g_*Solobacterium*, g_*Oribacterium*, g_*Mogibacterium*, g_*Atophobium*, and g_*Lachnoanaerobaculum*. Other noticeable increase was observed in f_*Sphingobacteriaceae*, f_*Enterobacteriaceae*, f_*Coriobacteriaceae*, and f_*Erysipelotrichaceae*. In remission phase, differentially abundant bacteria were mostly identified from the orders *Neisseriales, Fusobacteriales*, and *Rhodobacterales*. o_*Neisseriales* showed the highest enrichment in contrast with their abundance in active phase and control (Figure 4C). The dataset used for performing differential abundance analysis is provided in Supplementary Table 3.

### Functional Characterization of the Sample Categories

We further performed the functional characterization of the data using PICRUSt which identified 41 and 241 metabolic pathways according to level 2 and level 3 KEGG pathway classifications, respectively. The level 2 KEGG pathways showed that pathways involved in membrane transport, replication and repair, amino acid metabolism, carbohydrate metabolism, and Translation were the five most highly abundant pathways. Other most abundant pathways included energy metabolism, metabolism of cofactors and vitamins, nucleotide metabolism, cellular processing and signaling etc. In contrast, the least abundant pathways included nervous system, immune system, circulatory system, excretory system, and cardiovascular diseases. All of the 41 level 2 pathways were identified from the three categories of samples. However, differential abundance analysis using LEfSe, identified 14 pathways which were significantly enriched in the three different categories. For instance, pathways for carbohydrate metabolism, nucleotide metabolism, metabolism of terpenoids and polyketides and enzyme families were significantly more enriched in the active phase of the disease. In contrast, pathways for lipid metabolism and cellular processing and signaling were remission phase and healthy controls, respectively (Supplementary Figure 5B).

**Figure 5:**
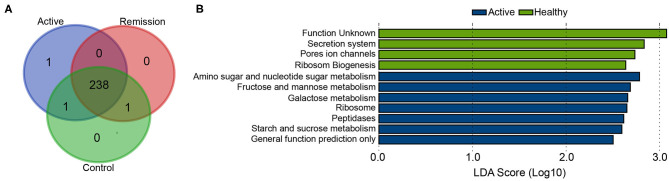
Differentially abundant functional categories among active phase, remission phase, and healthy controls identified using LEfSe. **(A)** Venn diagram showing common functional pathways in three sample groups. **(B)** Significantly differentially abundant pathways in active phase of CD and healthy cases.

KEGG classification level 3 showed presence of 241 different functional categories. 238 of these pathways were commonly present in all three categories of the sample (Figure 5A). Majority of the genes were classified to the pathways of transporters, general function prediction only, DNA repair and recombination proteins, ABC transporters, ribosome, purine metabolism, and pyrimidine metabolism as the most highly abundant pathways among all samples. Differential abundance analysis using LEfSe (LDA > 2.5) identified 11 differentially enriched pathways; seven were enriched in the active phase of CD whereas other four were enriched in healthy controls whereas no pathway was significantly enriched in the remission phase. The highest enrichment was observed for the pathway including genes with unknown function (KEGG pathway: Function unknown) with LDA > 3. All other significant enrichments were supported by LDA > 2.5 and <3. Amino sugar and nucleotide sugar metabolism, fructose and mannose metabolism, and galactose metabolism showed the highest enrichment among the metabolic pathways in active phase, in contrast with the healthy controls. Control samples were also enriched in pathways secretion system, pores ion channels, and ribosome biogenesis (Figure 5B).

## Discussion

Crohn's disease (CD) is a type of Inflammatory bowel disease (IBD) that involves several factors that contribute to its pathogenesis, including genetic as well as environmental factors. These factors could indirectly affect microbiome of the oral cavity and gut which in turn may increase the incidence of the disease (Lira-Junior and Figueredo, 2016; Agossa et al., 2017). Gut microbiome has been studied extensively in IBD and its disturbance is well-known to occur during the disease. Dysbiosis in the oral microbiome can also cause alterations in the immune regulation and therefore participate in the pathogenesis and development of IBD. Here, we analyzed the oral microbiome in Chinese CD patients during different phases of disease to improve our understanding of the oral microbiome and their association with CD. Salivary samples were obtained during active phase and remission phase of CD as well as healthy controls from the patients enrolled at the Ruijin Hospital, Shanghai (Affiliated with School of Medicine, Shanghai Jiao Tong University, Shanghai, China). Our results indicated that, during the active phase of the CD, α-diversity was significantly decreased in contrast with remission and healthy control. The number of observed species also decreased significantly in the active phase of the disease. The differences in remission and healthy controls were not significant in terms of Chao1 and Observed Species (Figures 1A,B). These results are in concordance with the previously published study by Xun et al. (2018) that highlighted lower α-diversity during the CD in contrast with the oral microbiome from the healthy samples. Similarly, β-diversity using unweighted and weighted UniFrac distances also indicated significant differences among these samples. Healthy samples clustered with samples from remission phase whereas most of the oral microbiome samples from the active phase of CD clustered separately. These results are similar to a recent study (Xun et al., 2018) that showed that β-diversity was significantly lower in salivary samples obtained from active phase of CD.

In majority of the samples from active phase of CD, we observed depletion of *Neisseria, Haemophilus, Fusobacterium* and *Porphyromonas*. Said et al. (2014) also reported the depletion of *Neisseria* and *Haemophilus* in the salivary microbiome of the IBD patients (Said et al., 2014). Furthermore, we also observed noticeable differential abundance between healthy controls and active phase samples in terms of OTUs classified as p__*Proteobacteria*, f_*Pasteurellaceae*, c_*Gammaproteobacteria*, p__*Alloprevotella* and g__*Desulfobulbus*. Among these, several bacteria e.g., *Alloprevotella* play a critical role in maintaining pH in the oral cavity and key for normal functionality of the oral cavity. Furthermore, f__*Veillonellaceae*, c__*Negativicutes*, p__*Actinobacteria*, g__*Pedobacter*, g__*Salmonella*, g__*Prevotella*, f__*Bacteroidetes*, and p__*Bacteroidia* were enriched in the Active phase of the disease in our samples. A recent study on the role of salivary microbiome in causing IBD (both UC and CD) revealed higher abundance of *Veillonellaceae* in CD, hence, the higher abundance of *Veillonellaceae* in our study is consistent with their finding (Xun et al., 2018). Furthermore, *Salmonella* includes facultative, Gram-negative bacteria that can infect several hosts including humans. However, Salmonella infection in the gut is known to have potential involvement in causing IBD. Determination of their role in oral microbiome during the active phase of CD requires validation by performing further studies. *Prevotella* are among the dominant genera in the oral cavity and strong drivers of the dysbiosis of oral microbiome during IBD. Hence, there higher abundance in the active phase of CD in our study concords with these findings.

Furthermore, we also performed comparative analysis of the functional categories among the three sample types. Our results indicated enrichment of seven different functional categories in the active phase, in contrast with the other two categories, which mostly included pathways of metabolism. Among the depleted pathways in the active phase, noticeable pathways included ribosome biogenesis, pores ion channels, and secretion system. Impaired ribosome biogenesis has recently been shown to impair muscle growth in murine model of IBD (Figueiredo et al., 2016). The depletion of this pathway in our study is consistent with this observation by Figueiredo et al. (2016).

Our study has several limitations. We did not collect fecal samples from the patients, a comparison of oral and gut micrbiome during different phases of the disease would be interesting to perform in the near future. Furthermore, we could not collect longitudinal samples from the same patients during active and remission phases of CD, which can be performed in the future work to highlight microbial dynamics within same patients during the course of the disease.

## Conclusions

In conclusion, our study provided insights into the taxonomic and functional diversity and variations between the two phases of CD i.e., active and remission phase in contrast with the healthy controls. We highlighted significant decrease in microbial diversity during the active phase of disease and identified several taxa and functional categories that are potentially biomarkers of the active phase of the disease. Although our results could be used to identify risk and severity of CD, further experimentation is required for comprehensive understanding and validation of these results.

## Data Availability Statement

The raw sequencing reads are accessible from NCBI SRA database using accession number PRJNA612402 (https://www.ncbi.nlm.nih.gov/bioproject/PRJNA612402).

## Ethics Statement

The studies involving human participants were reviewed and approved by Department of Gastroenterology, Ruijin Hospital Affiliated to Shanghai Jiao Tong University School of Medicine, Shanghai, China. The patients/participants provided their written informed consent to participate in this study.

## Author Contributions

ZW and LC provided designed and supervised the study. TZ, MRK, and JZ performed the analysis and wrote the manuscript. LH and CZ helped in the collection of the samples and clinical data from the patients. All authors read and approved the manuscript.

## Conflict of Interest

The authors declare that the research was conducted in the absence of any commercial or financial relationships that could be construed as a potential conflict of interest. The reviewer XT declared a shared affiliation with the authors to the handling editor at the time of review.
