# Supplementary material for: Dynamics of the Salivary Microbiome During Different Phases of Crohn's Disease
Source: Front Cell Infect Microbiol. 2020 Oct 6;10:544704. doi: 10.3389/fcimb.2020.544704 (PMC7574453; doi:10.3389/fcimb.2020.544704)
Supplement: Supplementary file 1 [file Table_1.DOCX]

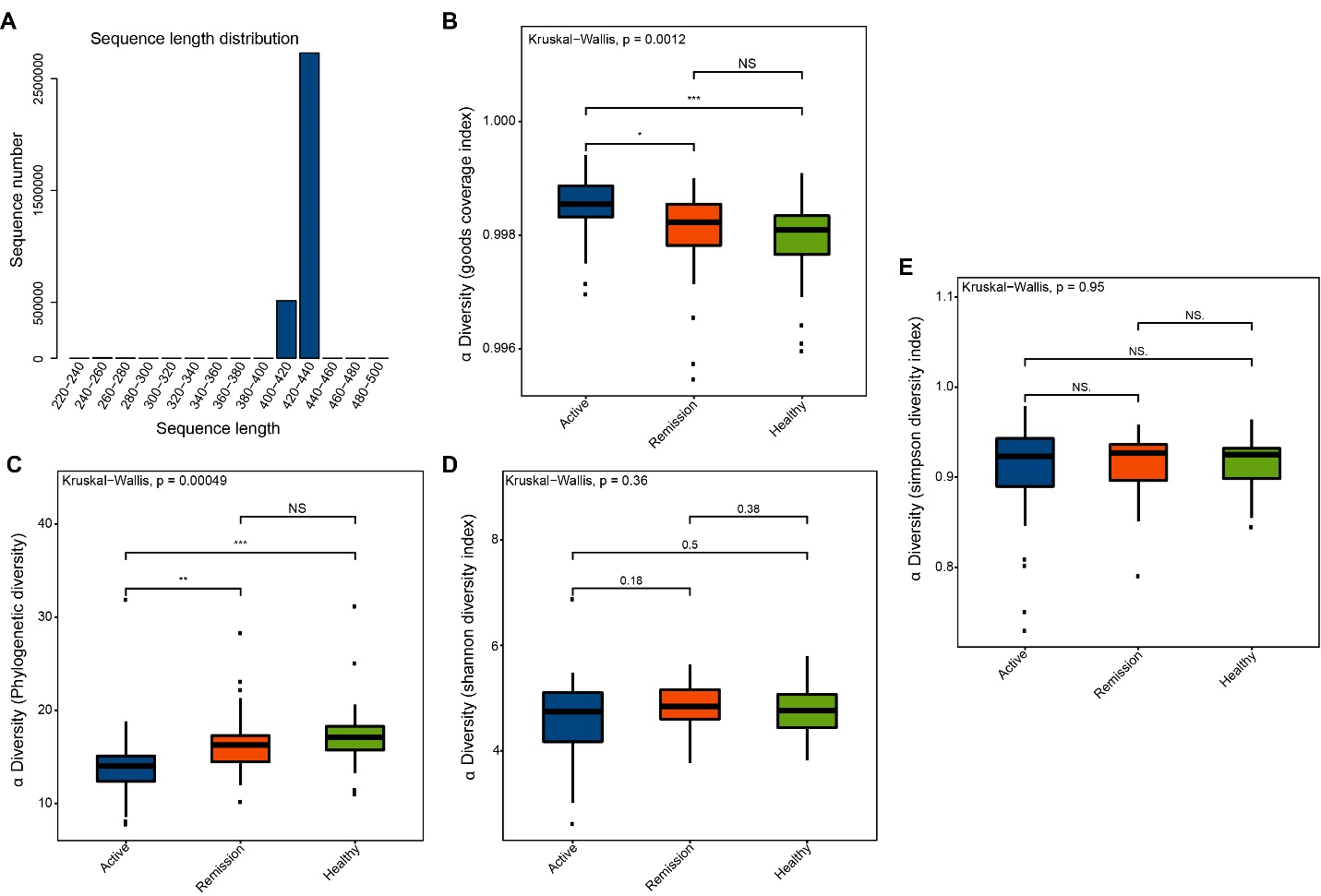


**Supplementary Figure 1:** Sequence length distribution and multiple alpha diversity indices in the Oral microbiome samples.


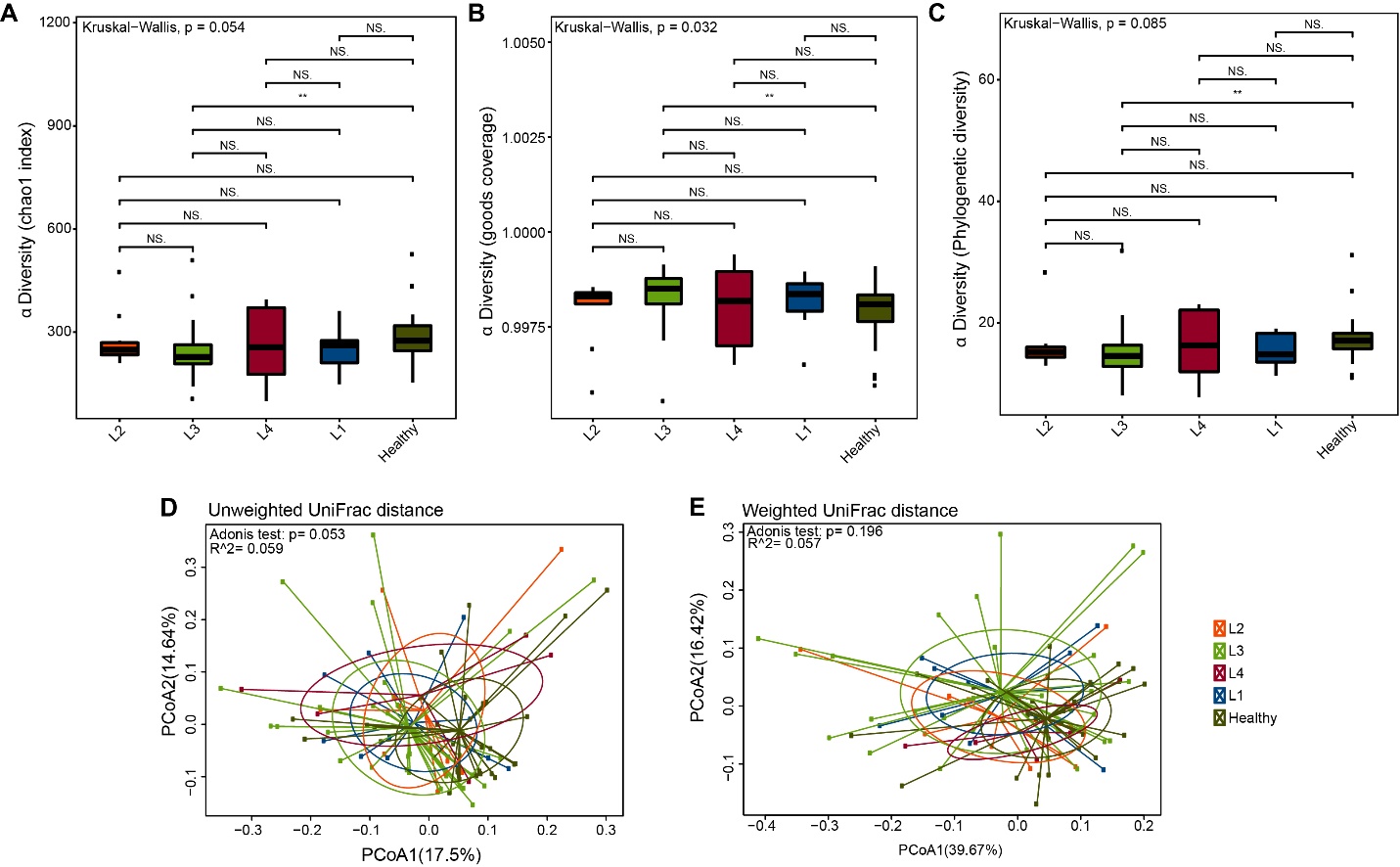


**Supplementary Figure 2:** Alpha and beta diversity indices in the Oral microbiome samples according to their Montreal classifications.


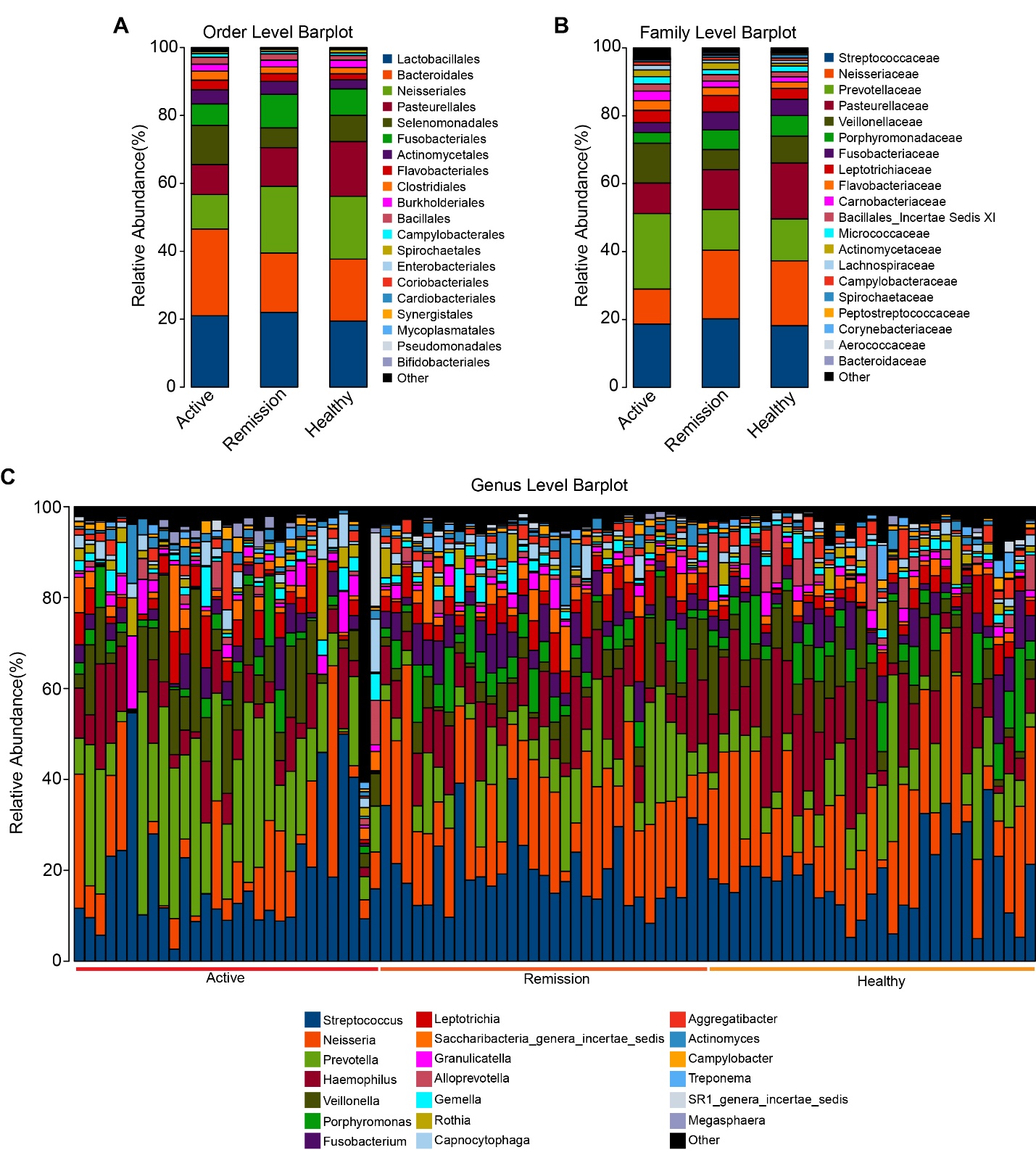


**Supplementary Figure 3:** Taxonomic distribution of the Oral microbiome samples at Order, Family and Genus level.


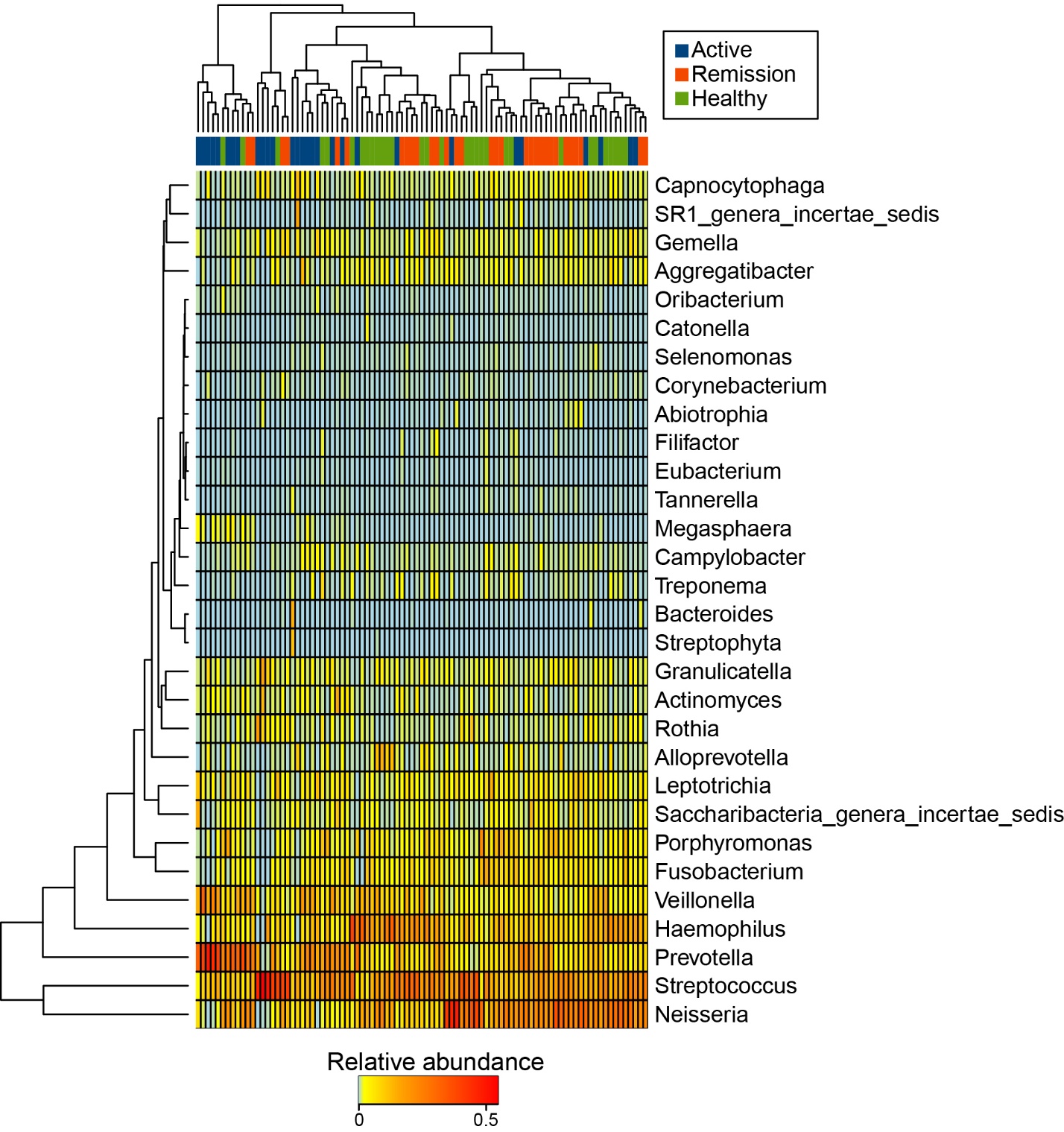


**Supplementary Figure 4:** Heatmap of the 25 most abundant taxa at Genus level


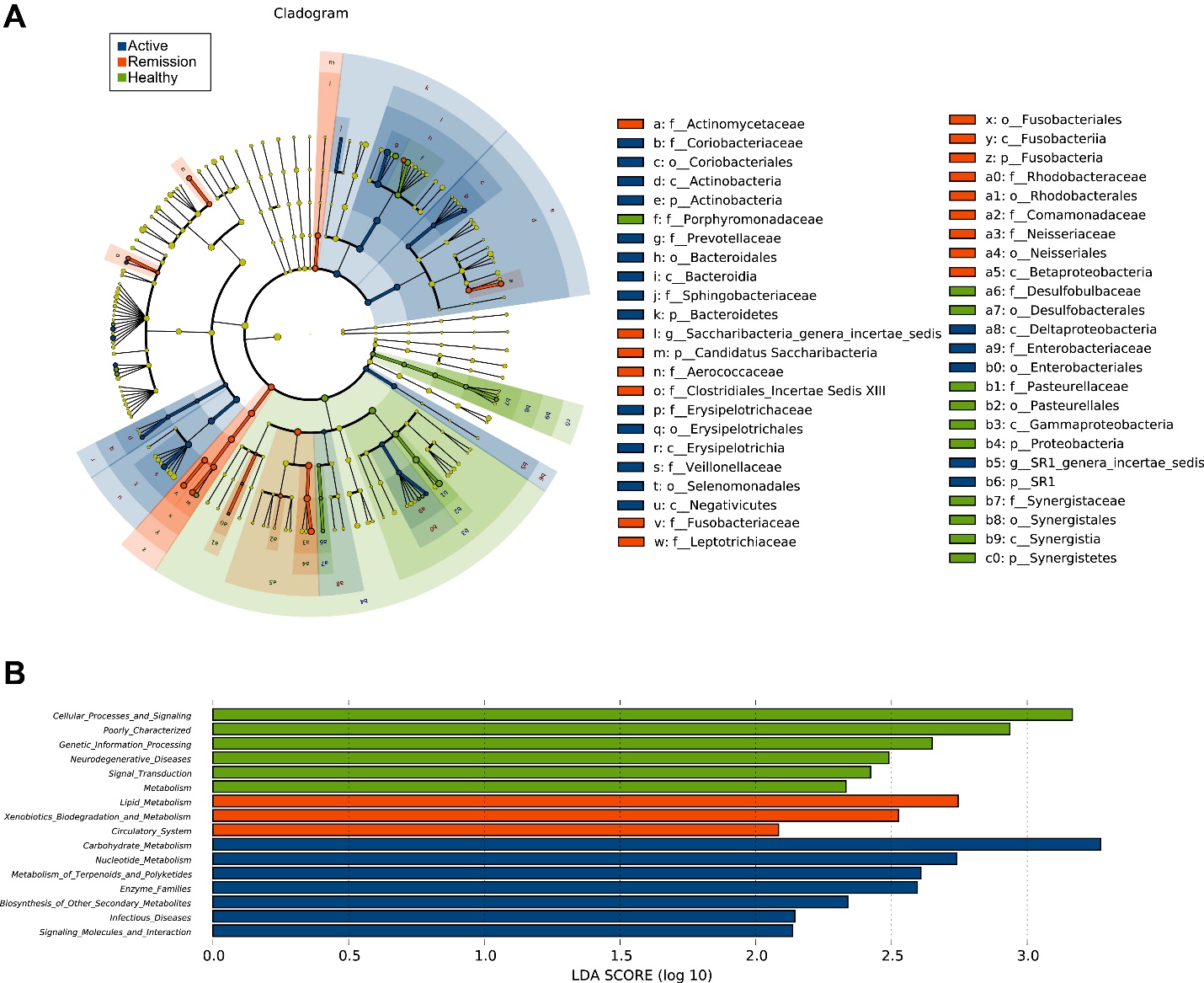


**Supplementary Figure 5:** Differentially abundant taxa and functional categories identified using LefSe.
